# “So I call myself healthy”: a qualitative study on health perceptions and healthcare experiences in older adults with multimorbidity

**DOI:** 10.1186/s12875-025-03010-w

**Published:** 2025-10-15

**Authors:** Klas Ytterbrink Nordenskiöld, Christina Sandlund, Caroline Kappelin, Karin Norman, Caroline Wachtler

**Affiliations:** 1 Department of Neurobiology, Care Sciences and Society, Division of Family Medicine and Primary Care, Karolinska Institutet, Huddinge, SE-141 83 Sweden; 2 Academic Primary Health Care Centre, Stockholm Region, Stockholm, SE-113 65 Sweden; 3 Department of Social Anthropology, Stockholm University, Stockholm, SE-114 18, Sweden

**Keywords:** Multimorbidity, Aged, Qualitative research, Primary health care, Person-centered care, Holistic health

## Abstract

**Abstract:**

An ageing population and its association with a rising prevalence of co-existing multiple chronic conditions poses increasing challenges for healthcare systems worldwide. In line with the World Health Organization ambition for societies to develop integrated care models and person-centered care, this study aimed to investigate what health means for individuals managing multiple chronic conditions and how these patients navigate a healthcare system primarily designed for single-disease management.

**Methods:**

A six-phase reflexive thematic analysis was conducted on 16 individual interviews with patients aged 67 to 87 years old in a Swedish primary care setting.

**Results:**

Two themes were developed. Firstly, *Resiliently Positioning as Non-Sick* that centered on how participants employed internal strategies to position their identity on the non-sick side of a health spectrum, and secondly, *Placing Yourself in the Hands of Healthcare,* which focused on the mixed feelings towards interacting with healthcare.

**Conclusion:**

Older individuals with multiple conditions tend to identify as non-sick and strive for autonomy. Engaging with healthcare can pose a threat to both their autonomy and their non-sick identity. They desire healthcare that works holistically, focuses on health and function, and avoids stigmatizing terms like multimorbidity. We recommend that policymakers and healthcare providers integrate this understanding and support for autonomy and holistic approaches into their efforts to deliver person-centered care.

**Supplementary Information:**

The online version contains supplementary material available at 10.1186/s12875-025-03010-w.

## Background

Healthcare systems worldwide aim to enhance patient outcomes by effectively identifying and treating diseases. However, the aging population and its association with a rising prevalence of multimorbidity [1, 2], commonly defined as the co-existence of multiple chronic conditions [2–5], presents significant challenges to conventional healthcare models. Prevalence estimates of multimorbidity vary widely due to inconsistent definitions, but studies suggest that up to 90% of adults aged 65 and older [2] in primary care settings may be affected [6], underscoring the widespread nature of the issue. While the most common definition of multimorbidity relies on a count of two or more chronic conditions, more nuanced definitions—such as those distinguishing complex multimorbidity [7] or emphasizing clinical relevance [5, 8, 9]—have been proposed to better capture the condition’s complexity. Despite their potential, these definitions have not yet been widely adopted in the literature [7].

Multimorbidity is linked to increased mortality [1], diminished quality of life [1, 7–9], polypharmacy [1], higher healthcare costs, and greater use of both primary and secondary care services [10]. It is also linked to engagement with multiple care providers and a higher risk of unplanned emergency visits and hospital admissions [11, 12]. While multimorbidity is also prevalent before the age of 65 [1], and many studies include both younger and older individuals [2], research shows increasing prevalence and higher mortality rates among older populations [1], highlighting the need for special consideration of this group.

Traditional healthcare approaches often prioritize a single-disease focus [13]. Yet, such disease-centric models may not adequately address patients’ personal health goals or improve their overall well-being [14]. Patients with multimorbidity face fragmented care, where multiple isolated treatment regimens and guidelines are combined without addressing patient overall well-being, risk for harmful treatment combinations, or personal goals in relation to life expectancy [14, 15]. They often self-manage to maintain function and prevent decline [16], facing challenges like uncertainty, reduced self-trust, and an increasingly limited lifestyle—challenges that are particularly pronounced among older individuals [17].

Two key areas of research have emerged in the effort to improve care for people with multimorbidity. First, a growing body of qualitative studies on patients’ experiences with healthcare [18]. These studies often examine how patients navigate fragmented systems, manage complex treatment regimens, and seek continuity and holistic approaches in their care [18, 19]. Second, there has been a recent development of clinical guidelines specifically addressing multimorbidity, primarily targeting older adults in primary care [20]. These guidelines focus on identifying vulnerable patient groups, avoiding inappropriate prescribing, and supporting individualized decision-making and follow-up [20]. However, they often lack practical recommendations for real-world implementation [20]. In parallel, interventions designed for older patients with multimorbidity have shown positive effects on outcomes such as mortality and cognitive function [21].Yet, they appear to have limited impact on health-related quality of life [21, 22], suggesting a disconnect between clinical priorities and what patients value most. This highlights the need for healthcare providers and researchers to better understand what truly matters to patients living with multimorbidity.

Aligning with the World Health Organization’s call for integrated care models [3, 23]—particularly for older adults with multimorbidity [24]—and to address a gap in the literature on how patients conceptualize multimorbidity, this study explores two key questions: What does health mean for older individuals managing multiple chronic conditions? And how do they navigate a healthcare system primarily designed for single-disease management? Addressing these questions is critical for aligning healthcare objectives with patient-centered care.

## Methods

In this qualitative study, we employed a six-phase reflexive thematic analysis, as outlined by Braun and Clarke 2022 [25], to identify and develop themes and subthemes from interviews with primary care patients.

### Approach to the research

In addition to their roles as researchers, the authors brought diverse professional perspectives to this study. Three of them, KYN, CK and CW, were actively practicing primary care physicians. Their perceptions of good patient care were shaped by years of interpreting the concerns of older individuals within the disease-oriented framework of the current Swedish medical system. They were also aware of the interpretative priority they had over what constitutes illness and disease. One author, CS, was a district nurse who for some time had focused primarily on research and teaching. CS brought not only medical perspectives but also theory from nursing science around conceptualizations of health. KN, a professor emerita of social anthropology, brought social, cultural as well as feminist and class related perspectives to the analysis. KN joined the team when initial familiarization with the material had just begun. CS and CK joined the team during the last phase of interviews. The authors ages ranged from 38 to 77 years old, and one was raised in the US.

Epistemologically, the authors adopted a subjectivist approach. This meant they viewed language as a good tool for understanding and describing reality—but not claiming this reality to be objectively true. Ontologically, they mainly positioned as interpretivists and saw truth as a result of subjective interpretations influenced by context, culture, and social dynamics – in contrast to a positivist position from which truth is the result of unbiased, objective measurements [26].

### Setting/participants

Individuals aged 65 and older with multiple chronic conditions were recruited to explore their experiences of health and primary care. The original aim was to examine how patients define the concept of multimorbidity. However, as the research progressed through engagement with the literature, discussions, and development of the interview guide, it became clear that multimorbidity is a term primarily rooted in the language of the healthcare system—not the patients themselves. To meaningfully explore the concept, we realized it was necessary to first understand how patients view health and experience the system that applies this label. This insight led us to broaden the study’s aim.

Initial participants were recruited from the control group of a previous intervention pilot study, where they had received usual care. Inclusion criteria were age 65 or older, at least two chronic conditions, increased care needs and ability to speak and understand Swedish or English. Exclusion criteria were ongoing care relationship with any member of the research team, or dementia.

After the first interviews, we identified a need for greater sociodemographic diversity. To address this, we expanded recruitment to two additional suburban primary healthcare centers in Stockholm—chosen for their socioeconomic and cultural diversity—and updated the study information to highlight our interest in patients born outside Sweden, patients with no education beyond high school, and patients receiving home care. These centers were selected using a mix of purposive and convenience sampling, based on existing research team connections.

**Fig. 1:**
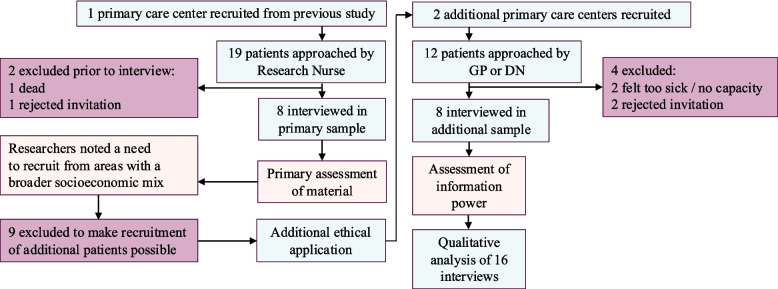
Recruitment process

In total, 31 individuals were invited in three steps. First, general practitioners (GPs) or district nurses (DNs) external to the study invited eligible patients with at least two chronic conditions during routine consultations, and distributed verbal and written information. For the first 19 patients, initial interest was followed up by a research nurse, and for the remaining 12, initial interest was followed up by KYN. Second, interested patients could ask questions and receive further information by phone from KYN. Third, those who agreed provided informed consent to KYN before the interview. Invitation was made between November 2022 and November 2023. The first eight interviews were conducted between January and February 2023, and the remaining eight between September 2023 and January 2024. Figure 1 describes the recruitment process.

### Dataset generation

A multiple-choice survey of basic sociodemographic data and use of Homecare was conducted before commencement of audio recorded interviews to initiate the conversation and get to know the participants. One-to-one interviews were performed to ensure integrity of participants and interview depth. Interviews lasted between 23 and 66 min, with an average duration of 44 min, and were recorded in their entirety. Interviews were transcribed in detail by a transcription bureau. KYN performed an initial reading and pseudonymization, corrected by simultaneous listening parts where the bureau had misspelled, misheard or misunderstood what the patient said. Participants were offered interviews in their homes, at their primary care center, digitally, or over the phone. Eight interviews were conducted at home, and eight at their primary care center. A semi-structured topic guide (Additional file 1) was used to ensure coverage of pre-formulated topics while allowing flexibility to explore topics raised by participants. All interviews were conducted by KYN. The number of all registered diagnoses, both acute and chronic during the last 3 years, and prescribed medications during the last year, were collected from electronic medical records after each interview, also controlling that participants met the inclusion criteria of two chronic conditions. Chronic conditions were defined based on a previous multimorbidity study [1].

### Characteristics of participants

Participants mean age was 78 years and they had a mean number of 20 diagnoses and 12 medications (Table 1).

**Table 1:** Basic demographics and medical information of participants

Number of participants	16 (100%)
Sex (female)	8 (50%)
Mean age (min—max) years	78 (67–87)
Marital status	
Alone	6 (37.5%)
Living apart together	1 (6.25%)
Cohabitant/Married	9 (56.25%)
Education level	
Elementary	4 (25%)
High school	4 (25%)
University college	8 (50%)
Country of birth (Sweden)	14 (87.5%)
Home care service (Yes)	5 (31.5%)
Mean number of diagnoses^a^ (min—max)	21 (10–33)
Mean number of medications (min—max)	12 (5–24)

^a^Total count of diagnoses, acute and chronic, during 3 years prior to interview

### Data analysis

A six-phase reflexive thematic analysis [25] was adopted to explore the interview material, guided by a Big Q ambition and an inductive, reflexive analytical approach. Big Q refers to a non-positivist stance, as outlined by Braun and Clarke, viewing knowledge as contextually situated and emphasizing the importance of reflexivity as a way to acknowledge and engage with subjectivity throughout the research process [27]. Data were mainly analyzed through digital handling, writing, rewriting, mind maps and illustrations of drafted themes and subthemes using software’s such as Microsoft Word and PowerPoint. Participants were not invited to the analysis phase of this study. All were invited to read their transcripts and provide feedback to KYN or CW if desired; two accepted the invitation, but none provided feedback. All authors familiarized themselves with the material through reading of all interviews. At two initial meetings, overarching ideas about the content were discussed and a primary codebook was formulated. KYN then coded all interviews using NVivo software version 14 for Mac [23] and produced approximately 150 codes that were clustered into four categories: Health, Disease/Illness/Multimorbidity, Healthcare, and Life/Death/Future. These clusters were brought to four analytical meetings for generation of initial themes and sub-themes. The analytical meetings followed a similar iterative process: KYN worked with the dataset to produce and refine analytical ideas and codes into themes and sub-themes, while the rest of the team performed reciprocal readings of one or two interviews each, giving input at meetings. CW gave additional input on analytical ideas between meetings. Team-meetings where themes and sub-themes were discussed, revised, and reformulated involved revisiting the material to refine codes. After the four analytical meetings, one overarching latent theme and nine sub-themes were decided upon and named, and the writing of the manuscript began. During the writing process, the nine sub-themes were first consolidated into four distinct sub-themes, and then after a fifth analytical meeting, further reorganized into two separate themes with a total of seven subthemes. KYN led the analytical meetings and drafted all text in the manuscript. Before finalizing the manuscript, the whole team had a final meeting to review the completed analysis. The process and writing adhered to the Reflexive Thematic Analysis Reporting Guidelines (RTARG) [24]. AI-assistance from Microsoft Copilot [28] was used to improve the readability and grammar of the text, as well as for the translation of the interview guide from Swedish to English.

## Results

**Fig. 2:**
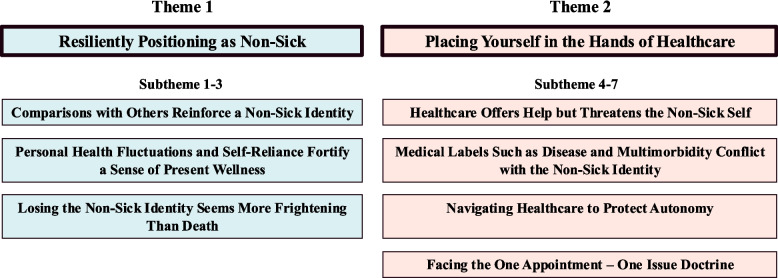
Themes and subthemes

Two major themes were formulated in the analysis: the first with three subthemes and the second with four subthemes. While distinct, the themes were closely interrelated. The first theme, *Resiliently Positioning as Non-Sick,* centered on how participants employed internal strategies to position their identity on the non-sick side of a health spectrum. The second theme, *Placing Yourself in the Hands of Healthcare,* centered on the mixed feelings towards interacting with healthcare. See Fig. 2 for a theme and subtheme overview.

### Theme 1: Resiliently positioning as non-sick

One of the strongest impressions from this material was how informants positioned themselves on the non-sick side of a health spectrum. We interpreted this positioning as an act of resilience—an active process of constructing and sustaining a positive self-perception of health despite having multiple chronic conditions and medications. Experiences of previously being sick positively influenced individuals’ present sense of wellness. Thus, the non-sick position appeared as dynamic, shifting to new equilibrium states in relation to current abilities and demands. From a medical perspective, this non-sick position was somewhat surprising. Individuals classified in health care with multiple diseases and as having multimorbidity actually remained relatively unaffected by these classifications. Diagnostic codes, it seemed, were not incorporated into patient’s identities and the positioning exemplified how health can be conceptualized whilst having multiple chronic conditions. Conceptions about patients being, by definition, sick were challenged through this analysis and diagnostic codes remained as examples of how the medical system classified patients, not as truths about how patients saw themselves. This theme is further developed in the following three subthemes.

#### Subtheme 1: Comparisons with others reinforce a non-sick identity

The non-sick position was based on a sense of wellness. However, this sense of inner wellness was not the sole factor behind this positioning. It also stemmed from comparing one’s health to the perceived wellness of others of similar age. Such comparisons were a common behavior observed throughout this material, involving various aspects of health and disease. Health in this context, appeared as a relative concept:

*Respondent 6, male, 86 years old: “I would say healthy, yes, and happily healthy, and I almost become religious (laughs) when I see out here the frequency of walkers, the frequency of canes and crutches, and also a group that I think has some dementia issues who wander around at 11 o’clock and come and wander around here.”*

Norms and ideas about what constitute a real disease, and why such definitions didn’t apply to one’s own condition, also seemed to influence the sense of wellness. A binary perception of health, as either having it or being sick, might be an underlying force to distance from conditions identified as real diseases.

*Respondent 16, female 69 years old*: *“There are people who have other conditions that are much worse. If you think about lung problems or being in a wheelchair or, well… which might be worse.”*

*Respondent 2, female 68 years old: “Yes, a typical illness might be cancer, diabetes, those kinds of lung diseases and such.”*

#### Subtheme 2: Personal health fluctuations and self-reliance fortify a sense of present wellness

Present wellness was influenced by awareness that life could be much worse. Informants knew they had health because they knew what it was like not to have it. In contrast to the external comparisons of subtheme 1, this content revolved around strictly personal experiences:

*Respondent 12, male 83 years old: “Because I lay there for a month and couldn’t get up and so on. I had diapers and I couldn’t get up to do my hygiene or anything. I was completely knocked out. Before I got these medications. Then everything changed.”*

Activities leveled with abilities and self-reliance for daily challenges formed reassurances of normality which highly influenced present wellness.

*Respondent 12, male 83 years old: “I feel really good. It’s nice to go to bed in the evening. Sometimes I stay up at night when I can’t sleep and have a sandwich. I watch some TV, stay up for a couple of Hours. I sleep about 5–6 h at night, but I can also nap during the day. I’m not tired, I try to engage in things I find fun. On the phone or on the tablet or on the computer.”*

*Respondent 9, male 80 years old: “Now I go and do a bit of shopping down there. We have home care for the shopping, but I like to get a newspaper every morning, little things like that. Now I can do it (…). So, I must say, I feel really good.”*

Attitudes towards ableness, of doing things yourself, remained important also under an increasing interference of illness on life:

*Respondent 15, female 87 years old: Yes, I’ve been involved all the time* (in my pursuits) *fixing and arranging things myself, so it has stuck with me. (…) So, I prefer to do everything. Not everything, I must admit, but you get used to it.”*

#### Subtheme 3: Losing the non-sick identity seems more frightening than death

When asked about the future, informants spoke about a fear of losing important aspects of the non-sick identity rather than a fear of dying. When death was mentioned, it was mostly as a statement of something that will eventually happen:

*Respondent 1, male 79: “Yes, it´s almost done. Won’t live forever. (…) But I’m at the age where I’m in the risk zone. It’s just a fact.”*

Focus was instead directed towards what could be done to remain non-sick:

*Respondent 1, male 85: “(…) That means that eventually, you will die, but it’s not something I think about directly. It will come when it comes. The only thing you can do about it is to try to stay active and keep moving. And somewhat, eat and drink in a somewhat healthy way, so to speak.”*

Naming feelings surrounding one’s own death is, in our experience, somewhat uncommon in the cultural context of this study. The absence of such emotions in this material therefore doesn’t necessarily mean that informants don’t have them. However, what we understood as fears about the future was the loss of important aspects of the non-sick identity:

*Respondent 5, male 80 years old: “For me, what I fear the most is being bedridden.”*

Loosing autonomy seemed to be the most frightening future, and the fear of not being able to advocate for one’s needs:

*Respondent 7, female 72 years old: “But there will come a day when you might not be able to speak up, so… it’s a bit scary.”*

### Theme 2: Placing yourself in the hands of healthcare

Another dominant impression we gathered from this material was the mixed feelings participants expressed about interacting with the medical system. These interactions involved putting oneself in the hands of healthcare, which could evoke both positive and negative emotions. While some aspects of healthcare were reassuring and helpful, others were ambiguous, negative, frustrating, or disappointing. To highlight the complexity of these interactions and their relationship with the non-sick self, we formulated the following four subthemes.

#### Subtheme 4: Healthcare offers help but threatens the non-sick self

Healthcare was seen as both support and a threat to the non-sick position. All interactions seemed to involve at least some elements of both positive and negative feelings. The support provided by healthcare for some concerns was strong:

*Respondent 6, male, 86: “Yes, the gist was that I felt a bit embarrassed for having started so much, but for me, it’s a resource. I see it as a production department, like, damn, now I’ve unlocked that machine to do that job.”*

We understood seeking care as a process where informants acknowledged a potential disease and placed themselves in the hands of healthcare. This situation included feelings of fear, dependence, and loss of control. Since the non-sick position was founded on wellness, self-reliance, and autonomy, these feelings were problematic. By postponing a healthcare interaction, these negative feelings could be avoided:

*Respondent 7, female 72 years old: “And then she* (the doctor*) says, but if you thought it was throat cancer, why did you wait so long? Then I have no sensible answers, I can’t, no, you should hurry to call, but then it becomes the opposite, it becomes too dangerous, then you don’t call.”*

However irritating it could be when ailments added up, the burden of the solution had to balance with the cost for the non-sick life:

*Respondent 3, male 75 years old: “And then I’ve just felt that there are too many surgeries, I can’t do all of this at once, I need to have a life too (laughs), not just deal with illnesses. But none of this is life-threatening or very critical, but it’s damn irritating when they all add up like this.”*

Negative feelings from healthcare interactions could arise when medical observations were not in line with patient needs and could be interpreted as criticism:

*Respondent 2, female 68 years old: “I don’t know, there was a period when I always heard about my… my weight. Eventually, I said that I get anxiety when I go there. Why? Because I don’t always want to hear how much I weigh, that I’m too fat. Because I know it myself without having to be told all the time.”*

Some illnesses such as pain appeared more difficult to seek help for than others and could leave informants in a state where they felt disbelieved and without support. That someone else got to define what was sick and what was not included a loss of power and threat to the non-sick position. This could cause further reluctance to seek care:

*Respondent 2, female 68 years old: “It eventually makes you not seek help when you really should. (,,,) Because it’s so easy to feel like you’re more believed to have, well, imaginary illness, you know? (…) That you’re a hypochondriac or something like that. (…) It’s because it’s pain that can’t be seen, it doesn’t show up in tests, but you have it, it just exists there, you know. And then eventually you don’t seek help, you just wait.”*

#### Subtheme 5: Medical labels such as disease and multimorbidity conflict with the non-sick identity

Despite living with chronic conditions for several years, medical labels such as disease and multimorbidity conflicted with the non-sick identity. Societal norms and a lifelong perception of being primarily healthy likely influenced this conflict. These medical labels were perceived negatively and caused cognitive dissonance, making acknowledgment reluctant. However, since these labels were a mediator to receiving medical aid, informants noted the need to acknowledge them. After receiving medical aid and experiencing improved health, it became possible to distance from these labels once again.

*Respondent 9, male 80 years old: “Yes, I don’t know how to say it. Of course… There were diseases, and with that came medical help and so on. Otherwise, you don’t get medical help. I had to go to both the southern hospital, as I said, and Karolinska hospital. I’ve been to the primary care center countless times.”*

Self-derived terms for living with multiple concurrent chronic conditions were not clearly expressed by the informants, and they reacted rather negatively on this question:

*Respondent 16, female, 69 years old: “So you want to put me in a box?”*

Being categorized was undesired and had negative connotations. Multimorbidity, a term commonly used in both clinics and academia, was perceived as a misclassification and incompatible with the non-sick position.

*Respondent 7, female 72 years old:* *“And when she* (the nurse) *said that* (multimorbidity*), I thought, why is she saying that? I don’t have that (…) But I didn’t say anything, I just thought it. (…).*

*Interviewer:* *Yes, so tell me about this word* (multimorbidity)* or how you perceive it.*

*Respondent: Yes, but then it’s very bad, then you are both old and sick and miserable, and I am neither old, miserable, nor sick (laughs).”*

The “sick” position implied by the term multimorbidity was associated with dependence, loss of autonomy, and a diminished sense of self-worth. Feeling of being a lost cause captures the emotional meaning of the word multimorbidity in this context:

*Respondent 1, male 85 years old: “Yes, well, a person with multimorbidity, it’s a bit… partly, it’s a bit negative too, of course. It almost seems like a wreck. I don’t really feel like a complete wreck, I don’t think I do.”*

*Respondent 10, female 75 years old: “(…) Then it’s over.”*

#### Subtheme 6: Navigating healthcare to protect autonomy

To protect autonomy and strengthen feelings of control in interactions with healthcare, knowledge about the system was essential. Knowing who to contact, how to contact them, and when to best contact them, counteracted feelings of dependence and powerlessness, and supported a position on the non-sick side of the spectrum.

*Interviewer: “You say that you are satisfied with the contacts here and that you find it easy to get appointments, is that correct?”*

*Respondent 14, female 72 years old: “Because I know the system.”*

Navigating the system also involved negotiations and advocating your needs:

*Respondent 8, female 75 years old: “But she* (the district nurse) *stood her ground, and I stood mine, so eventually she had to give in. Because I said, I have the right to talk to my doctor. Yes, but she is so… she is so rarely here, so… yes, but I said, a phone appointment, I’m not asking for an in-person appointment, I said. I’m asking for a phone appointment. Then it was very easy. It happened within a week.”*

Awareness about healthcare working from priorities that sometimes conflicted with one’s own was beneficial. The ability to find the right person and argue your case became a means of upholding autonomy:

*Respondent 1, male 85: “It took a while to convince her that I should have one of these sensors, but eventually, it worked out. (…)*

*I1: You had to argue your case?*

*R1: Yes, well, it costs money.”*

When healthcare became too authoritarian, autonomy could also be upheld by courage to clearly speak up and set boundaries.

*Respondent 2, female 68 years old: “(…) It was a bit difficult at first. You had to muster up the courage because I was raised to… back when I was little, you weren’t allowed to address a doctor informally. That still lingers a bit, this thing about authority and such. But once I did it, it felt good.”*

#### Subtheme 7: Facing the one appointment – one issue doctrine

A “one appointment – one issue” rule seemed common when interacting with healthcare, sometimes leaving informants frustrated and disappointed. While there might have been times when discussing one concern was sufficient, informant’s shifting state of health often created a need to address multiple concerns. After a health-worsening event, a new set of concerns would be added to an already extensive list. Hospitals emerged as resourceful providers of aid for acute and threatening conditions, but the old ailments were often left unaddressed.

*Respondent 7, female 72 years old: “So, if you put it this way: When you go for one problem, you have a sore throat and go to the health center and so on, well, there’s not much to say about that. But in the beginning when I came home, yes, first I was in the hospital, but when I came home, because you get help with the worst part at the hospital and that’s good. But there are all the other ailments that they don’t care about, or at least they don’t have the time or the possibility. Then you have to seek out your healthcare center, different instances so to speak, and in the beginning, I couldn’t even get to the health center.”*

Under the circumstance of constantly managing multiple issues, the one issue rule added obstacles in upholding a non-sick position:

*Respondent 3, male 75: “What I start with is what we manage to get through. The rest we don’t have time for, he* (the primary care physician) *looks at the clock and – unfortunately, I can’t talk to you any longer now. When can I get the next appointment then? Well, in about three months.”*

Even though some examples, primarily from the experience of district nurses, highlighted another way of working, where having someone who listened provided feelings of relief and support:

*Respondent 11, male, 79 years old: “Then I brought up all these things that I had been thinking about* (with the district nurse)*. Because it’s difficult to book a doctor and go through everything. Usually, you can only talk about one thing at a time. Now I took everything at once that I had. Old issues that I wondered about. And we went through them.”*

## Discussion

This qualitative interview study of older individuals with multiple chronic conditions illuminated resilient strategies to position oneself as non-sick, as well as the dual perceptions towards interacting with healthcare. The importance of maintaining a non-sick identity was underscored by the fear of losing it, with healthcare acting as both a facilitator and an aggravator in this effort. Participants expressed a desire for holistic approaches and a focus on health and autonomy as potential facilitators.

### Resiliently positioning as non-sick

The concepts of “actually feeling quite healthy” [29], maintaining autonomy and control [30], and positioning oneself as non-sick despite chronic illness [31] have been previously described in other populations. In contrast to younger individuals, older adults have been proposed to be better equipped to manage chronic illness, either because they anticipate it or have developed coping skills over a lifetime [32]. This non-sick positioning relates to sociological descriptions of a healthy self, which can be explained through perceptions of normality and engagement in normal behaviors [33]. We found that engagement in and self-reliance on activities that matched one’s capacity were important for maintaining a healthy identity. Notably, greater capacity for such activities has been linked to a greater sense of health [34].

In contrast, descriptions of populations with multimorbidity often suggest they suffer from disease-engulfing processes, leading to a disconnection from the foundations of a healthy self [30] and experiencing a diminishing world when faced with diseases [17]. This rather negative perspective is common in clinical practice and resembles the disablement process, which describes disability acquired late in life as a sum of pathologies that reduce capacity relative to societal demands [35]. Our analysis challenges this downhill view of health with chronic disease in late life. Instead of a slope towards functional decline, dependence, and a sick identity, individuals seem to move dynamically on the health spectrum, finding new equilibrium states of being non-sick in relation to their abilities and demands.

Incorporating this understanding of a self-perceived healthy ageing into clinical practice is one of the main implications of this study. We believe this could be facilitated by a basic understanding of concepts such as the biopsychosocial model [36], the shifting mindsets in late life described through gerotranscendence [37], and taking into account the quite vast literature and strong emphasis by patients with multimorbidity on moving away from a single disease focus to a holistic approach that includes several aspects of their health [38].

The fear of losing the non-sick position was emphasized in our analysis and relates to previous work on the fear of an unpredictable future, increased dependence, and powerlessness when living with advanced care needs [39]. Studies on fear of decline are often disease- or symptom-specific [40, 41]. However, in our study, this fear seems more generic. Fear of death was not clearly expressed in our interviews. Notably, earlier studies indicate that people generally strive to sustain hope despite the inevitability of death [42], and that the fear of it appears relatively low in the young-old population, who experience a period of life with relatively good function, purpose, and capacity for daily activities [43].

### Placing yourself in the hands of healthcare

Placing oneself in the hands of healthcare is a crucial process described in our analysis, involving seeking aid while acknowledging the power dynamics involved. Asymmetries in information and power during clinical encounters, as well as the challenging process of prioritizing health concerns between physicians and patients with complex care needs, have been highlighted previously [44]. Multimorbidity and polypharmacy augments this challenge by increasing the need for information sharing during consultations. When prioritization fails, patients may feel overwhelmed or abandoned [44]. The failure of clinical decision-making for complex care needs, and inadequate support from single-disease guidelines during time-limited consultations, are also noted by others [45].

In our interviews, the term “multimorbidity” induced significant tension. We believe this tension reflects both a distancing from such medical labels as previously described [29], and a lack of widespread understanding of the term. Stigma, often defined as an attribute that is deeply discrediting or being labeled with socially disqualifying characteristics [33], may also be involved. The negative connotations to the word “multimorbidity” that we observed may be an expression of such stigma in the Swedish context. For successful implementation of patient-centered care models focusing on multimorbidity [3, 46] and healthy ageing [47], we propose two key strategies: First, acknowledge the tension that arises when patients are labeled as ill despite identifying as non-sick. This calls for open dialogue around the term multimorbidity and the exploration of alternative, less stigmatizing language—such as “person with multiple conditions”—alongside efforts to build consensus on acceptable terminology. Second, promote awareness of the concept of multimorbidity and its clinical value in shifting away from a single-disease framework toward more holistic approaches. After all, this shift reflects a broader movement, one that is being called for by both patients [19, 38, 48] and other stakeholders [49–52].

The ability to navigate the healthcare system while protecting autonomy was highlighted in our analysis. This skill, which closely relates to the concept of health literacy [53], is not equally possessed by everyone seeking care but is highly influenced by factors such as age, minority status, income, and education [54]. Higher healthcare use is also associated with greater difficulties in navigation, especially for those lacking social capital [55]. Hence, the challenges of maneuvering in healthcare likely causes different levels of frustration and disengagement from care for different groups. Taking this into account to facilitate availability and equal access to care is important when designing policy, routines, or interventions for future healthcare.

Finally, we interpreted seeking primary healthcare with multiple concerns as potentially very challenging, especially for invisible concerns such as pain. Higher age and psychosocial problems are previously known to be associated with a need for longer consultations [56], and the doctors ability to manage multiple concerns is linked to both higher quality of care and lower costs [57]. Despite this, a “one appointment – one issue” rule appeared to be common from the experience of our informants, leading to frustration and long waiting times for subsequent consultations. Finding innovative ways to schedule appointments [58], as well as supporting doctors’ communication skills to effectively address the patient agenda [59], have been highlighted as promising strategies to tackle these challenges.

### Clinical and research implications

The significance of the “non-sick self” as we explored in this study, and its connection to engagement in normal activities, supports a more holistic approach to care, shifting the focus beyond purely medical concerns to what truly matters to patients. Additionally, the study highlights two important considerations: the variation in patients’ ability to navigate the healthcare system, and the need to address power dynamics in patient–provider interactions. Incorporating these insights into medical training may enhance the shared decision-making process central to patient-centered care and help ensure more equitable access to services. While the term multimorbidity appears as highly undesired by participants in this study, we do not advocate for its full abandonment. Rather, we recommend increasing awareness of its implications and value, considering alternative terminology, and applying the concept thoughtfully in the development of guidelines that address multiple conditions simultaneously, moving away from the “one appointment, one issue” model.

### Strengths and limitations

Strengths of this study include the diverse composition of the research team, with variation in age, academic backgrounds, and medical professions. Additionally, the study offers a rich, first-hand account of how multimorbidity can be conceptualized through the experience of health and healthcare interactions for primary care patients with multiple chronic diseases—an area that has been underexplored in previous research.

The authors believe that this analysis may be relevant, and transferable to other settings. However, certain considerations should be kept in mind when interpreting and applying these results. During recruitment, two patients declined invitation due to feeling too sick, and one patient died. This unplanned exclusion of acutely ill, or unstable, participants might have influenced the findings.

The interviews were conducted in Swedish and it’s possible that the Swedish translation of the word multimorbidity was interpreted differently than it might have been in another sociolinguistic context.

The population studied were all above the age of 65 and mainly of Swedish descent without disabilities from birth. Information on ethnicity was not included due to its classification as sensitive personal data in Sweden, which would have required specific justification and ethical approval. Gender differences were not considered in the analysis. Furthermore, distinctions between older and younger individuals with multimorbidity, as well as the influence of socioeconomic factors, remain underexplored in much of the existing literature on multimorbidity [60] and were not addressed in this study. Given that living with a disability can affect how people access and receive care [61], and that being non-white [62] or identifying as LGBTQ + [63] can also shape these experiences, such factors should be taken into account when interpreting the findings of this study.

Information on psychiatric and psychological diagnoses was not obtained from participants’ medical records, nor were the number of chronic diseases beyond a simple count of two or disease severity assessed. This is a limitation, as the findings may be influenced by the severity of the participants’ conditions. Additionally, common mental health problems can increase the risk of various physical conditions [64, 65], lead to greater healthcare utilization [66], and negatively impact quality of life [67]. These factors should be considered when interpreting the results.

The absence of participant involvement in the analysis phase may be considered a limitation. To address this, the authors aimed to incorporate diverse perspectives within both the data and the analytical team, considering factors such as professional background, country of birth, health status, and age.

## Conclusion

Older individuals with multiple conditions tend to identify as non-sick and strive for autonomy. Engaging with healthcare can pose a threat to both their autonomy and their non-sick identity. They desire healthcare that works holistically, focuses on health and function, and avoids stigmatizing terms like multimorbidity. We recommend that policymakers and healthcare providers integrate this understanding and support for autonomy and holistic approaches into their efforts to deliver person-centered care.

## Supplementary Information

Additional file 1.

## Data Availability

The datasets generated and analyzed during the current study are not publicly available due to the detailed level of the interview material but are available from the corresponding author on reasonable request.
