# Supplementary material for: “So I call myself healthy”: a qualitative study on health perceptions and healthcare experiences in older adults with multimorbidity
Source: BMC Prim Care. 2025 Oct 15;26:312. doi: 10.1186/s12875-025-03010-w (PMC12522901; doi:10.1186/s12875-025-03010-w)
Supplement: Supplementary file 1 — Additional file 1. [file 12875_2025_3010_MOESM1_ESM.docx]

**Interview Guide**

**Presentation of Researchers**

**Information about the Study**

The reason we want to conduct this study is that people over 65 years old, who have more than one disease, are the largest patient group in primary care. It is a diverse group with different backgrounds, diseases, and experiences. The purpose of this study is to hear patients' thoughts and experiences of having multiple diseases. Everything said will be recorded and used for a research article. In the article, no response will be traceable to any specific person. If you want to stop during the interview, just let us know, and you don't need to explain why. The interview will take about 30–60 minutes.

Do you have any questions before we start?

**Topic 1: Introduction**

Can you tell me about your medical history, that is, what diseases or ailments you have?

What is troublesome about these diseases? How do you notice them? Which ones do you notice?

What is significant about having multiple diseases at the same time, as opposed to just one at a time?

Can you describe a situation when you felt more ill than usual? What happened then?

**Topic 2: Concepts for Multiple Diseases**

In healthcare, we meet many people with multiple diseases at the same time. We are considering what might be good terms to use to describe them.

How would you refer to a person who has multiple diseases or ailments?

In healthcare, the terms multimorbidity or multimorbid are often used for people with multiple diseases. How do you perceive these terms?

Can you describe a person that you perceive has mulitmorbidity?

If you were described as having multimorbidity, how would you feel about it?

**Topic 3: Contacts with the Health Center**

I understand that you have (disease/ailment X, Y, and Z).

How do you experience seeking or receiving care for your diseases and ailments?

Is there a difference between having one or multiple diseases or ailments when you seek care? How do you notice it?

What affects how much help you need when you seek care?

**Topic 4: Conclusion**

Now we have talked about your diseases, the term multimorbidity, and how it is for you to seek care. Is there anything else you think I should have asked about this?

How was it to participate in this interview?

Is there anything else you want to add before we finish?
